# A bidimensional measure of empathy: Empathic Experience Scale

**DOI:** 10.1371/journal.pone.0216164

**Published:** 2019-04-29

**Authors:** Marco Innamorati, Sjoerd J. H. Ebisch, Vittorio Gallese, Aristide Saggino

**Affiliations:** 1 Department of Neuroscience, Imaging and Clinical Sciences, Institute of Advanced Biomedical Technologies (ITAB), G. d'Annunzio University of Chieti-Pescara, Chieti, Italy; 2 Department of Human Sciences, Università Europea di Roma, Rome, Italy; 3 Department of Medicine and Surgery, Unit of Neuroscience, University of Parma, Parma, Italy; 4 Institute of Philosophy, School of Advanced Study, University of London, London, United Kingdom; 5 School of Medicine and Health Sciences, G. d’Annunzio University of Chieti-Pescara, Chieti, Italy; Universita degli Studi di Udine, ITALY

## Abstract

Empathy is a key notion in the study of sociality. A phenomenological perspective on empathy as intersubjective understanding offers a common ground for multiple dimensions. Corresponding to the dichotomy between perceptual and cognitive levels, two constructs can be distinguished: vicariously experiencing and intuitively understanding others’ emotions. We developed and validated a new questionnaire for the assessment of individual differences in empathy. In a first study (N = 921), we created a questionnaire measuring empathy consisting of a pool of 75 items. Exploratory factor analysis suggested to retain two factors (“Intuitive Understanding” and “Vicarious Experience”), whereas a 30-item version of the questionnaire had satisfactory psychometric properties. In a second study (N = 504), we administered the 30-items questionnaire and several concurrent/divergent measures. Confirmatory factor analysis indicated that a two-factor structure best represented its latent factor structure. The results show that our questionnaire could be considered a reliable and valid measure of empathy with internal consistencies of 0.93 and 0.95 for Vicarious Experience and Intuitive Intuitive Understanding, respectively. Whereas our questionnaire mostly showed the expected convergence with existing scales of empathy, the correlations also suggest that it adds valuable new information to the assessment of empathy. The two-factor structure suggests that the perceptual (vicarious) experience and the basic (non-effortful) cognitive awareness of others’ emotions can be assessed as distinct constructs. This bidimensional structure that distinguishes between the perceptual experience and the basic cognitive awareness of others’ emotional states connects theoretical, empirical, and clinical data from psychology and neuroscience.

## Introduction

### Empathic experiences: Intersubjective understanding

Empathy is a key notion within the study of sociality that originates in philosophy and phenomenology [1–6]. Notwithstanding the vast and enduring attention dedicated to empathy in literature for more than a century, no consensus has been reached about its exact definition and measurement. Consequently, until today, the term has been inconsistently used to describe various interpersonal phenomena within as well as between disciplines including psychology, philosophy and neuroscience [7–12]. Nevertheless, following a more traditional phenomenological account, the description of empathy might be generally summarized as a multifaceted function that consents an individual to experientially connect with others’ inner experiences and to be naturally aware of those experiences [8,13,14,4,15], while also recognizing that these experiences primarily originate in the other [7,16,17]. In other words, it concerns the intersubjective understanding of others’ experiential life.

In current psychological and neuroscientific literature, interrelated constructs corresponding to these experiential and understanding aspects of empathy can be identified that have received thorough attention [9,12,18]. Firstly, the construct of vicarious experience concerns the phenomenon of intersubjective emotion, which consists of the participation in the emotion of another individual through affective, vicarious responses to the other’s emotional state [9,13,14]. Secondly, the awareness of another individual’s mental experiences, which entails the conscious understanding of the emotions of another individual is denoted by the construct of social understanding [19–21].

From the perspective of intersubjectivity, empathy could thus be considered as a compound product of these constructs that reflect its perceptual and cognitive elements, respectively [13,18,21,22]. Accordingly, it has been proposed that it might not be adequate to measure individual differences in empathy psychometrically at a general level, but rather at the level of each of these single constructs independently [18], despite several authors also proposed total scores to present a single score for empathy.

Notably, although such perceptual and cognitive elements of empathy may approximate the distinction between emotional [23] and cognitive empathy [24,25], some relevant differences exist. For instance, emotional (or affective) and cognitive empathy commonly refer to more general emotional responses to those of another individual (including concern or sympathy as opposed to vicarious responses) and the effortful understanding of the emotions of another individual (based on cognitive inference rather than intuitively grasping them), respectively [9,20,21,24,25]. Another difference is that the concepts of emotional and cognitive empathy are rooted in different theoretical backgrounds (“simulation approach” versus “theory theory approach”) that are often viewed as either opposing or independent without providing a unified explanation. By contrast, to address the multidimensional notion of empathy, we depart from a common phenomenon of intersubjectivity [13] that constitutes a fundamental aspect of sociality throughout life [26].

### Measuring empathic traits

Historically, individual differences in empathic traits have been measured by self-report questionnaires (e.g., the Hogan's Empathy Scale [27]), usually from a multifaceted perspective tentatively differentiating between experiential/emotional and cognitive components. For instance, emotional aspects have been measured along with cognitive aspects of empathy in general measures of empathy (e.g., the Interpersonal Reactivity Index—IRI, the Basic Empathy Scale, BES, and the E-Scale [28,29–32]). In other cases, questionnaires have been constructed to measure specifically emotional aspects of empathy, including the Balanced Emotional Empathy Scale (BEES [33]) and the Toronto Empathy Questionnaire (TEQ [34]).

Nevertheless, questionnaires commonly used to measure empathy-related constructs generally share relevant theoretical and psychometrics shortcomings. Firstly, the different questionnaires referred explicitly or implicitly to different definitions of empathy and different theories at the construct level [27–29,35–37]. This could partly explain the fact that questionnaires putatively measuring empathy do not share the same factor structure and that correlations among scores of different empathy tests are generally weak. The same explanation could be valid for inconsistencies in neuroimaging studies investigating correlations of empathic traits [38]. Indeed, some authors noted the imprecision in the interdisciplinary use of psychological empathy concept due to a lack of connections with psychological theories and behavioural data [9]. This limitation is evident in empiric studies investigating their reciprocal convergent validity [39]. For example, the IRI is intended to measure four empathic dimensions (i.e., Perspective taking—PT, Fantasy–FS, Empathic Concern–EC, and Personal Distress—PD) and, although Davis [28] supposed that these dimensions could be integrated in the cognitive/emotional empathy bipolarity, other authors disputed his hypothesis [40]. Secondly, items composing these measures do not always discriminate between empathy and sympathy or emotional arousability [30,34,39,41–43]. This is evident from the study of Reniers et al. [44]. Instead of applying factor analysis as done for the TEQ [34], the authors constructed their scale (the Questionnaire of Cognitive and Affective Empathy, QCAE) selecting items from existing questionnaires (Empathy Quotient—EQ; HES, IRI; impulsiveness-venturesomeness-empathy questionnaire—IVE-7), after assessing their content validity. Among the 150 items included in those questionnaires, only 65 were considered valid measures of empathy, and only 36 were evaluated as measures of emotional empathy.

Thirdly, research frequently reported inconsistent results for the latent structure of the empathy questionnaires. The original factor structure of the questionnaires (IRI and EQ) or its unidimensionality (BEES and Emotional Contagion Scale—ECS) were seldom confirmed by successive independent studies [45–58]. Many studies investigating the factor structure of the IRI were not able to confirm its four-factor structure as originally proposed [49–57]. A study from Muncer & Ling [58] did not support the adequacy of the three-factor structure of the EQ. Moreover, although they obtained an internal consistency of 0.85 for the total EQ scale, multiple items showed not satisfactory item-total score correlations, whereas some items saturated on more than one factor [58]. Five dimensions have been identified for the BEES [59]. More recently, studies failed to confirm the factorial structure of the ECS. One study reported not completely satisfactory psychometric properties [60]. Some authors using confirmatory factor analysis suggested the fit of a five-factor model (factors derived from the five emotion categories) and a hierarchical two-factor model [61,62]. Also Kevrekidis et al. [63], lo Coco et al. [60], and Gouveia et al. [64] reported the fit of multidimensional solutions for the factor structure of the ECS. Even the BEES, ideally constructed to measure specifically emotional empathy [33] and to eliminate the limitations affecting its predecessor, the Questionnaire Measure of Emotional Empathy (QMEE [65]), has itself several limitations. Similar to the QMEE, the BEES is not a unidimensional measure of emotional empathy. Moreover, its dimensions don’t clearly relate to any theoretical structure of emotional empathy, and using the questionnaire to compute a total score could be misleading [33,48,59,66]. Furthermore, it is not clear whether all the BEES items tap onto emotional empathy or other constructs (e.g., “I easily get carried away by the lyrics of a love song”, “I don't get overly involved with friends' problems” [39]).

Fourthly, the reliability of the questionnaires under investigation may be questionable as well, especially for the IRI, making scores as well as correlations with other measures prone to instability [67].

These findings put doubts on whether existing scales are properly measuring the notion of empathy, how scales are theoretically related, and if comparisons between results from different countries and cultures are reliable. A factorial structure of questionnaires that not coincides with the theoretical structure of empathy is problematic for studies that aim at forcing the results from a test to converge in a single score, or perhaps more, as indicators of empathy based on scores on single items. These problems have not been resolved completely by psychometric instruments that were developed during more recent years (Empathy Assessment Index—EAI, E-Scale, TEQ, QCAE). In particular, it is not clear what aspects of empathy existing questionnaires exactly measure or whether they properly distinguish between different theoretically defined dimensions as independent factors. This makes their applicability uncertain, for instance, to correlate scores with behaviour, symptomatology or brain function and structure.

### Aims

The comprehensive literature described above casts doubts on whether existing questionnaires properly measure empathy and how tests and their theoretical underpinnings could be related to each other. In addition, no questionnaire has been developed to directly extricate and study individual differences in two major constructs rooted in the phenomenological tradition to assess empathy as intersubjective understanding: the bodily and sensory perception (i.e., Vicarious Experience) and the cognitive awareness (i.e., Intuitive Understanding) of others’ emotions.

In the light of the illustrated shortcomings, the present work aims to develop and validate a new questionnaire to assess individual differences in the established constructs associated with the notion of empathy: the Empathic Experience Scale (EES). For this purpose, we decomposed the construct into two relevant theoretically based aspects: Vicarious Experience and Intuitive Understanding. We present results from two different studies: in the first one we developed a new questionnaire to measure empathy, and in the second one we administered our questionnaire and several concurrent/divergent measures to confirm factor structure and investigate psychometric properties of the EES.

## Methods

### Participants

Participants were recruited through advertisements posted in public places and for established community groups. All participants participated on a voluntarily and anonymous basis. They received no honorarium for completing the assessment. Written informed consent was obtained from all participants after full explanation of the study procedure, in line with the Declaration of Helsinki and its revisions [68]. The local Ethics Committee approved the experimental protocol.

The construction sample (Study 1) included 921 Italian adults (449 men and 471 women; 1 missing data) aged 18+ years (mean age = 30.38; SD = 13.22). Sociodemographic characteristics of the sample are reported in Table 1.

The validation sample (Study 2) included 504 Italian adults (207 men and 294 women; 3 missing data) aged 18+ years (Mean age = 40.80; SD = 20.63; range = 19/89 years). Sociodemographic characteristics of the validation sample are reported in Table 1.

**Table 1:** Sociodemographic characteristics of the construction and validation samples.

	Construction sample *n* = 921		Validation sample *n* = 504	
Variables	Frequencies	Percentages	Frequencies	Percentages
**Age–**years	30.38*	(13.22; 18/65)**	40.80*	(20.63; 19/89)**
**Women**	471	51.1	294	58.3
**Marital Status**				
Not married	626	68.0		
Married	244	26.5		
Other	51	5.5		
**School attendance ≤ 8 years**	116	12.6	139	27.6
**Job status**				
Employed	299	32.4	191	37.9
Unemployed	71	7.7	0	0.0
Student	511	55.5	190	37.7
Other	40	4.4	123	24.8

Footnotes.

* Mean

**(Standard deviation; range).

### Item construction

The authors analyzed international literature on empathy and intersubjectivity as described in the introduction section, and defined two major properties:

1. The experience of vicarious sensorimotor or emotional states as a reaction to the emotional state of another individual, i.e. participating in someone else’s emotional state by experiencing similar emotions. This definition differentiates feeling *with* others (i.e. vicarious experience) from feeling *for* others (i.e. sympathy and compassion) [20,29,69], the latter being characterized by dissimilar feelings of concern or worry that emerge from others’ negative emotions [5,70,71]. Thus, we segregated vicarious experiences from sympathy to highlight the participation in others’ emotions as a unique reaction.
2. Intuitive Understanding, i.e., the (effortless) cognitive awareness of the emotional or sensorimotor state of someone else. This definition distinguishes understanding as a basic cognitive awareness of the mental states of others from understanding as an inferential or imaginative process (i.e., effortful understanding). Hence, we focus on intuitively grasping others’ emotions as a naturalistic and primary form of social understanding [13,69].

Subsequently, the 75-item questionnaire was administered to a convenient sample of 28 (19 women and 9 men) Italian adults (mean age = 44.36 years; SD = 16.69; range = 23/78 years) to investigate their comprehensibility and, depending on the results (when 3 or more participants rated the item as not comprehensible or only slightly comprehensible), they were modified to improve their level of comprehensibility.

### Measures

All the participants in the construction sample were administered a sociodemographic checklist assessing age, sex, marital status, school attendance, and the current job, and the pool of 75 items developed in the previous steps. Participants were asked to rate each item according to a Likert-type scale ranging from 1 (Not at all true) to 5 (Completely true).

All the participants in the validation sample were administered the sociodemographic form and a battery of questionnaires including the 30-items version of the EES, the Interpersonal Reactivity Index (IRI [28,29]), the Balanced Emotional Empathy Scale (BEES [33], The Teate Depression Inventory (TDI [72,73]), the State-Trait Inventory for Cognitive and Somatic Anxiety (STICSA [74]), and the Marlowe-Crowne Social Desirability Scale (MC-SDS [75]). A description of these questionnaires is provided in the Supplementary Material.

### Statistical analysis software

All the analyses were performed with the statistical software for social sciences SPSS 20.0 (Armonk, NY: IBM Corp.), Factor 10.3.01 [76], and Mplus 7.0 (Muthén and Muthén,1998–2010).

### Statistical analysis study 1

The number of items to retain in the questionnaire and its dimensionality were investigated via exploratory factor analysis. We used unweighted least squares (ULS) estimator on a polychoric matrix of correlations, with Promin rotation. Adequacy of the correlation matrix for factor analysis was investigated with the Bartlett’s test of sphericity and the Kaiser-Meyer-Olkin (KMO) test. Adequacy of the correlation matrix is suggested by a significant Bartlett’s test (*p* < 0.05) and a KMO index > 0.70. Hull’s method [77] was used to suggest the number of factors to retain. Only items with a loading ≥ 0.40 on a single factor were considered for further analyses.

Finally, to limit response burden without failing in fully capturing the construct [78–80], we decided to limit the maximum number of items included in the final version of the questionnaire to 30–50 and to include the same number of items in each dimension. Thus, we compared versions of the questionnaire with 30, 40, and 50 items for their internal consistency and the presence of problematic items (i.e. items for which the Cronbach alpha improved or did not change when the item was excluded from the questionnaire). The version of the questionnaire with the minimum number of items and satisfactory reliability (α > 0.80) was selected as the final version. Included were the first 30–50 items with the highest corrected item-total correlations.

### Statistical analysis study 2

Three factor models were tested and compared for their fit to the data: (a) one-factor model, (b two-factor model, and (c) bifactor model. The one-factor model corresponds to the view of a single empathy construct, the bifactor model to the existence of two constructs that are uni-dimensional in measuring a higher-level phenomenon, and the two-factor model to independent constructs that measure different aspects of sociality.

For the confirmatory factor analysis, we used a Mean-and Variance-adjusted Weighted Least Square (WLSMV) estimator with a polychoric correlation matrix. Model fit was assessed using the following indices: (1) the Root Mean Square Error of Approximation (RMSEA), with values between 0.05 and 0.08 indicating adequacy of the model, and values below 0.05 indicating evidence of absolute fit [81,82]; (2) the Tucker Lewis Index (TLI), with values ≥ 0.90 indicating reasonable fit of the model [83]; (3) the Weighted Root Mean Square Residual (WRMR), with values of less than 1.0 indicating good fit [84]; and (4) the chi-square (χ2) test and the normed χ^2^ (χ2/degrees of freedom). P-values for the χ^2^ test greater than 0.05 and a normed χ^2^ less than 5 [85] indicate that the model is an adequate fit to the data, although the χ^2^ test over rejects true models for large samples. For the bifactor model, loadings of items on the general factor (Empathy) and the two first-order specific factors (Vicarious Experience and Intuitive Understanding) should also be considered when assessing their fit to the data. In general, the bifactor model tests the presence of a strong general factor and the quasi-unidimensionality of the measure, which is supported only when the loadings of items are significant for the general factor as well as for the intended specific factor, and when the loadings of items on the general factor are stronger than those on the specific factor.

Exploratory structural equation modelling (ESEM [86]), which integrates both exploratory and confirmatory factor analyses, was used when no models demonstrated to fit adequately the data for exploring possible reasons of misfit of the models tested. We used a Mean-and Variance-adjusted Weighted Least Square (WLSMV) estimator with a polychoric correlation matrix. Model fit was assessed using the same indices used for the confirmatory analysis.

To evaluate the possible presence of differential item functioning (DIF) and lack of factor invariance, the fit of a baseline multiple indicators multiple causes (MIMIC) factor model with sex as covariate and containing no direct effects from sex to item responses was tested and compared with a final model that included a direct path from sex to items, which resulted directly associated with sex in a series of MIMIC models with single direct paths. Additionally, factor invariance was tested with a multi-group factor analysis, comparing a configural model (i.e., the same model is tested in both groups, but all the parameters may differ between the groups) with a model with metric/scalar invariance (i.e., the same model is tested in both groups, and all factor loadings/thresholds are constrained between groups).

McDonald’s omega was reported as measures of internal consistency of the EES. Pearson’s *r* coefficients were reported as measure of association with other questionnaires.

## Results

### Study 1

#### Exploratory factor analysis of the empathic experience scale

The correlation matrix was suited for factor analysis (Bartlett’s test of sphericity = 27481.1; degree of freedom, DF = 2775; *p* < 0.001; KMO = 0.93). The analysis resulted in 15 factors with an eigenvalue > 1 (eigenvalues ranging between 16.67 and 1.04) for an explained variance of 60.4%. However, the method of Hull [76] suggested to retain only 2 factors with eigenvalues of 16.67 (explaining 22.2% of the variance) and 7.21 (explaining 9.6% of the variance) explaining cumulatively 31.8% of the total variance.

Thirty items had significant loadings (between 0.41and 0.80) on Factor 1, while 26 items had significant loadings (between 0.40 and 0.72) on Factor 2 (see S1 Table). Only items assessing the predisposition of the observer to intuitively recognize the emotional state of the other loaded on Factor 1 and consequently it was labeled “Intuitive Understanding”. Items evaluating the predisposition of the observer to experience emotions isomorphic to the internal state of the other loaded on Factor 2 and was labeled “Vicarious Experience”.

#### Comparisons between versions of different length of the empathic experience scale

We compared three different versions of the questionnaire with 50, 40, and 30 items respectively. The 50-items version had satisfactory reliabilities (alphas of 0.94 and 0.91 for Intuitive Understanding and Vicarious Experience, respectively). However, partial alpha coefficients indicated that two items were problematic (deleting item #24 alpha coefficient for the dimension Intuitive Understanding improved from 0.937 to 0.938; deleting item #55 alpha coefficient for the dimension Vicarious Experience improved from 0.909 to 0.910). Shorter versions (with 40 or 30 items) both had satisfactory internal consistency (40 items version: alphas of 0.93 and 0.90 for Intuitive Understanding and Vicarious Experience, respectively; 30 items version: alphas of 0.91 and 0.89 for Intuitive Understanding and Vicarious Experience, respectively), and no problematic items. Thus, the 30-items version could better limit response burden, while maintaining satisfactory psychometric properties when taking internal consistency into consideration. Further analyses will consider this version of the questionnaire. The Italian version (S1 Questionnaire) as well as an English translation (by a native English speaker) (S2 Questionnaire) of the 30-items EES are provided in the Supporting Information.

The mean scores for the total sample were 52.63 (standard deviation = 9.03; range = 15/75), and 38.53 (standard deviation = 10.30; range = 15/68) for Intuitive Understanding and Vicarious Experience, respectively. There were no ceiling and floor effects (only 0.1% of the respondents reported the minimum possible score for Intuitive Understanding and Vicarious Experience, and 0.5% reported the maximum possible score for Intuitive Understanding).

### Study 2

#### Dimensionality of the empathic experience scale

Fit indices for the competing factor models are reported in Table 2. All the competing models had a significant χ^2^ test, and WRMR > 1, which suggests the possible presence of unmodeled factors. Also, other indices supported the conclusion of misfit for the one-factor model, but not for the two-factor and the bifactor models. Fit indices for the two-factor and the bifactor models were comparable, except for WRMR, which indicated a better fit of the bifactor model. Nevertheless, despite that all the items loaded significantly (*p* < 0.05) both on the general factor and one group factor, fourteen loadings (mostly from the Vicarious Experience dimension) on the general factor were < 0.40, and several items loaded more strongly on the group factors than on the general factor. Moreover, it also has been suggested that WRMR might provide misleading results [87], whereas the χ^2^ statistic is sensitive to sample size. These results indicate weak evidence for the presence of a general factor in the EES, and they also indicate that the two-factor model may represent the latent factor structure of the EES better than the bifactor model.

**Table 2:** Fit indices for the competing models for the empathic experience scale.

Model	χ^2^	χ2/degrees of freedom	RMSEA	95% CI	TLI	WRMR
**One factor**	5094.53***	12.58	0.158	0.154 / 0.162	0.70	3.92
**Two correlated factors**	1406.28***	3.48	0.073	0.069 / 0.077	0.94	1.66
**Bifactor model**	1203.06***	3.21	0.069	0.065 / 0.073	0.94	1.25

Footnotes.

***Significant for p<0.0001.

Considering all these results, the following analyses are focused on the two-factor model. All the items loaded significantly on the hypothesized factor (see S2 Table), and the two factors (Intuitive Understanding and Vicarious Experience) were weakly and positively correlated with each other (*r* = 0.37; *p* < 0.01; R^2^ = 0.10), indicating that the two scales may measure different constructs.

To improve the two-factor model, inspection of the modification indices > 4 suggested to include in the model some crossloadings. However, to gather more information about the possible causes behind the elevated WRMR value (e.g., the presence of unmodeled factors), we performed exploratory structural modeling with 3 and 4 factors. Both models still had significant χ^2^ test (3-factor model: χ^2^_348_ = 1214.04; *p* < 0.001; 4-factor model: χ^2^_321_ = 922.42; *p* < 0.001), and the 3-factor model also had WRMR > 1 (= 1.11; RMSEA = 0.073, 95% CI = 0.069 / 0.078; TLI = 0.94). By contrast, the WRMR and other fit indices indicated acceptable fit of the 4-factor model (WRMR = 0.90; RMSEA = 0.063, 95% CI = 0.059 / 0.068; TLI = 0.95).

Analyzing factor loadings for the 4-factor model, two major results were evident: (1) The first two factors corresponded to the original factors of the two-factor model as all the items included in the original Vicarious Experience and Intuitive Understanding factors were significant (*p* < 0.001) and their loadings were > 0.50 (Intuitive Understanding: between 0.64 for item 16 and 0.86 for item 24; Vicarious Experience: between 0.52 for item 23 and 0.81 for item 13). There were several significant (*p* < 0.05) crossloadings (4 for Intuitive Understanding, with loadings ranging between 0.07 for item 9 and 0.17 for item 27; 6 for Vicarious Experience with loadings ranging between 0.06 for item 20 and 0.09 for item 16), but most of them were < 0.10 and all were < 0.20; (2) The third and the fourth factors had only two items with loadings > 0.40 (items 15 and 17 for Factor 3 and items 20 and 26 for Factor 4).

Inspecting the content of each pair of items, it was evident that they were similar in their content (item 15 “Those who know me tell me that I am not able to distance myself from the sadness of others”, and item 17 “Those who know me tell me that I am very affected by the emotions of others”; item 20 “I am able to know intuitively that a person feels uncomfortable even when I am in a group of people”, and item 26 “I notice immediately if someone in a group feels uncomfortable”).

Thus, we re-run a confirmatory factor analysis for the two-factor model including residual covariances between the two couples of items. The models had better fit to the data than the original model (χ^2^ = 1081.42, *p* < 0.0001; χ^2^/degrees of freedom = 2.69; RMSEA = 0.060; 90% CI = 0.056/0.065; TLI = 0.96; WRMR = 1.41), although WRMR was still > 1.

McDonald’s omega was 0.93 and 0.95 for Vicarious Experience and Intuitive Intuitive Understanding, respectively.

MIMIC models containing a single direct path from sex to one item response indicated the possible presence of DIF for eleven items (*p* < 0.05; items no. 3, 5, 9, 11, 13, 15, 16, 20, 21, 26, and 29). Direct paths from sex to all these items were included in a final model (χ^2^ = 1061.35, *p* < 0.0001; χ^2^/degrees of freedom = 2.53; RMSEA = 0.057; 90% CI = 0.053/0.062; TLI = 0.96; WRMR = 1.38), which confirmed a significant parameter estimate for the direct paths of 7 items (items 3, 5, 9, 11, 16, 20, and 26), but not for other 4 items (13, 15, 21, and 29). Fit of a baseline MIMIC model containing no direct effects from sex to item responses (χ^2^ = 1115.03, *p* < 0.0001; χ^2^/degrees of freedom = 2.57; RMSEA = 0.058; 90% CI = 0.056/0.065; TLI = 0.96; WRMR = 1.41) was compared to the final model with direct effects from sex to item responses. The results indicated that the inclusion of direct paths from sex to item responses significantly improved the model (χ^2^ difference test = 72.39, DF = 11, *p* < 0.0001). Multigroup factor analysis indicated the fit of the configural invariance model (χ^2^ = 1455.46, *p* < 0.0001; χ^2^/degrees of freedom = 1.81; RMSEA = 0.059; 90% CI = 0.054/0.064; TLI = 0.96; WRMR = 1.68), but also indicated that constraining factor loadings between the groups significantly worsened the model (χ^2^ difference test = 54.66, DF = 28, *p* < 0.01. Model with metric invariance: χ^2^ = 1449.65, *p* < 0.0001; χ^2^/degrees of freedom = 1.74; RMSEA = 0.057; 90% CI = 0.052/0.061; TLI = 0.96; WRMR = 1.77). This means that a common factor structure exists for males and females, but that total scores for females and males on both dimensions of the EES (Vicarious Experience–females: mean = 42.45 and standard deviation = 10.63; Vicarious Experience–males: mean = 33.98 and standard deviation 9.76; Intuitive Understanding–females: mean = 53.66 and standard deviation = 9.64; Intuitive Understanding–males: mean 49.42 and standard deviation = 9.76) may not be directly compared as some items are not equally related to the latent factors across sex.

#### Correlations among psychometric measures

Table 3 lists correlations of the Vicarious Experience and the Intuitive Understanding subscales with convergent and discriminatory measures. Correlations among existing psychometric empathy measures (BEES and IRI subscales) are described in S3 Table. Vicarious Experience correlated significantly, and in the hypothesized direction, with all the dimensions of the IRI and BEES (with *r* ranging between |0.10| for the IRI PT and |0.64| for the BEES Sp and total score). Intuitive Understanding also correlated significantly, and in the hypothesized direction, with all the dimensions of the IRI and BEES (with *r* ranging between |0.14| for the BEES F3 and |0.37| for the BEES F2), except for the IRI PD (*r* = -0.02). Thus, the EES dimensions were moderately to strongly correlated with convergent measures of empathy, with higher coefficients for Vicarious Experience. The BEES and the IRI were also weakly to strongly associated showing correlations from |0.08| (*p* > 0.05) between BEES F1 and IRI PD to |0.74| (*p* < 0.01) between the BEES total scores and IRI EC.

**Table 3:** Correlations among measures.

	Empathic Experience Scale	
	Vicarious Experience	Intuitive Understanding
Empathic Experience Scale—Vicarious Experience		0.37**
**BEES**		
**F1**	-0.29**	-0.18**
**F2**	0.53**	0.37**
**F3**	-0.60**	-0.14**
**F4**	0.52**	0.25**
**F5**	-0.34**	-0.25**
**Sp**	0.64**	0.33**
**Sn**	-0.47**	-0.20**
**BEES total score**	0.64**	0.31**
**IRI**		
**Fantasy**	0.51**	0.22**
**Empathic Concern**	0.49**	0.30**
**Perspective Taking**	0.10*	0.27****
**Personal Distress**	0.49**	-0.02
**MC-SDS**	0.16**	0.12**
**TDI**	0.18**	-0.08
**STICSA somatic**	0.36**	0.06
**STICSA cognitive**	0.34**	0.05

Footnotes. BEES = Balanced Emotional Empathy Scale (F1-F5 subscales); Sp = BEES Positive items; Sn = BEES Negative items; IRI = Interpersonal Reactivity Index (4 subscales); MC-SDS = Marlowe-Crowne Social Desirability Scale; TDI = Teate Depression Inventory; STICSA = State-Trait Inventory for Cognitive and Somatic Anxiety.

Empathy is associated with lower scores on the BEES F1, F3, and F5 and higher scores on the BEES F2 and F4.

*Significant for p<0.05

**Significant for p<0.005

Both dimensions of the EES correlated significantly, but weakly, with social desiderability (*r* = 0.16 for Vicarious Experience and *r* = 0.12 for Intuitive Understanding). Vicarious Experience, but not Intuitive Understanding, was also significantly, but weakly, correlated with depression (*r* = 0.18; *p* < 0.01) and trait anxiety (somatic anxiety: *r* = 0.36; cognitive anxiety: *r* = 0.34, *p* < 0.01).

The BEES and the IRI were weakly correlated with social desiderability (BEES: from |0.12| for F3 to |0.34| for F2; IRI: from |0.03| for FS to |0.35| for EC) and had several significant, but weak, associations with depression (BEES: |0.09| for F5 and |0.19| for F3; IRI: |0.24| for FS and |0.26| for PD) and anxiety (BEES: from |0.12| for F1 to |0.24| for F3 for somatic anxiety, and from |0.11| for F2 to |0.23| for F3 for cognitive anxiety; IRI: from |0.14| for FS to |0.36| for PD for somatic anxiety, and from |0.09| for EC to |0.45| for PD for cognitive anxiety).

## Discussion

The aim of the present study was to develop and validate a new questionnaire measuring empathy by adopting a bidimensional perspective that distinguishes between experiential and cognitive aspects of intersubjective understanding. In study 1, we found that a 30-item version of the questionnaire had good psychometric properties (alphas > 0.89) and a two-factor structure. On Factor 1 loaded items assessing the predisposition of the observer to intuitively recognize the emotional state of the other (“Intuitive Understanding”), and on Factor 2 loaded items evaluating the predisposition of the observer to perceive emotions similar to the internal state of another individual (“Vicarious Experience”). This factor structure was confirmed in a new independent sample. In Study 2, the two-factor model had a better fit to the data than two competing models (i.e., one-factor and bifactor models). Although fit indices for the two-factor and the bifactor models were comparable or even slightly better for the latter, several items loaded more strongly on the group factors than on the general factor. These results indicate weak evidence for the presence of a general factor, thus favoring the two-factor model for the EES.

The finding of a bidimensional structure of empathy also connects theoretical insights, and basic and clinical empirical data concerning intersubjectivity and social cognition. For instance, the two-factor structure suggests that the perceptual experience and the cognitive awareness of others’ emotions constitute phenomena that can be psychometrically measured as distinct constructs. In accordance, the two factors differently correlated with measures of convergent validity (BEES and IRI). This implies that they reflect different cognitive and neuro-functional processes underlying interpersonal relations.

In addition to a theoretical point of view, the Vicarious Experience and Intuitive Understanding components are also relevant from a clinical perspective in cases of pathology in which the exact deficits in empathy and intersubjectivity remain a topic of discussion (e.g., [88,89–94]) and constitute possible targets of intervention, for instance, in the context of stress contagion [95] and suicide prevention [96]. Consistent with the detected two-factor structure of the EES, Coll et al. [18] proposed that emotional empathy might be the outcome of the two processes of Vicarious Experience and Intuitive Understanding of someone else’s emotions. These two processes might be differently affected in distinct pathological conditions.

### The factor structure of empathy

The observed two-factor structure of the EES is more interpretable than the rather fragmented structure reported for the BEES, the only questionnaire that specifically focuses on measuring emotional empathy [33,48,59,66], and than the structure of the IRI, which might be the most widely used questionnaire for measuring empathy [35]. Indeed, the IRI, measuring interpersonal reactivity more generally, has a multidimensional factor structure which cannot be easily integrated in existing empathy models [40].

The complex factor structure of the BEES and the IRI could also be an effect of a poor selection of items included in the questionnaires. Firstly, the different questionnaires referred explicitly or implicitly to different definitions of empathy at the construct level [27–29,35–37]. Accordingly, correlations between the BEES subscales and the IRI empathic concern and personal distress subscales (theoretically closests to emotional empathy) strongly vary (i.e., fluctuating between weak and strong). Secondly, several authors [30,34,39,41–43] suggested that these questionnaires also have items that confuse predisposition for empathy with other related constructs (e.g., sympathy, emotional control or arousability, or imagination). This could partly explain the fact that correlations among scores of different empathy tests are generally weak [39]. The same explanation could be valid for inconsistencies in neuroimaging studies investigating correlations of empathic traits [38]. Indeed, some authors noted the imprecision in the interdisciplinary use of the notion of empathy due to a lack of connections with psychological theories and behavioral data [9].

This situation becomes even more evident from the results of the study by Reniers et al. [44] who assessed content validity of items from existing questionnaires. Their results considered only 65 out of 150 items (and 36 for emotional empathy) as a valid measure of empathy. These biases could also be considered partially responsible for the low to moderate correlations found in our study between the EES and convergent measures of empathy. Indeed, only for the BEES we found strong correlations with our questionnaire (*r* > 0.60).

### Convergent and divergent validity

Supportive of the construct validity of our questionnaire was the fact that the correlations of the Vicarious Experience factor were stronger for the BEES, considered a specific measure of emotional empathy as affective responses to other individuals, than for the IRI. Furthermore, when considering only the correlations between our questionnaire and IRI subscale perspective taking, considered by Davis [28] as a measure of cognitive empathy, had weaker correlations, while empathic concern and personal distress had higher correlations, especially regarding the EES Vicarious Experience dimension. Congruent with the experiential aspect of this construct, BEES items and the IRI empathic concern and personal distress subscales mainly concern affective responses and experiences, but not understanding of others mental states. The IRI perspective taking scale, indicated as a measure of cognitive empathy, only weakly correlated with the EES. However, considering the distinction between effortful and intuitive forms of understanding, it could be argued that the IRI perspective taking scale falls in the former group, whereas our Intuitive Understanding factor falls in the latter group. Moreover, correlation coefficients for the fantasy scale, also considered a measure of cognitive empathy [28], were comparable to those of empathic concern and personal distress. However, we have to note that also the correlation between the BEES total score and the fantasy scale had a similar size, and previous studies suggested that the fantasy scale also might be linked with affective aspects of empathy [97].

Comparing the correlations of the EES Vicarious Experience and Intuitive Understanding dimensions more generally, correlations among measures thus suggest that BEES items better tap onto what is measured from the dimension Vicarious Experience of our questionnaire, while both BEES and IRI poorly tap onto what is measured from the dimension Intuitive Understanding. This suggests that the Intuitive Understanding dimension, while being a theoretically relevant dimension that, different from effortful understanding, reflects an aspect of empathy which is not being captured by the existing questionnaires. Particularly, the items on this dimension explicitly concern intuitive forms of understanding without relying on cognitive inference or effortful imagination: this is opposed to effortful or cognitive forms of social understanding as addressed by most other questionnaires (e.g., IRI and EQ) and which are considered relevant for theory of mind and explicity mentalizing, rather than empathy as a more spontaneous awareness of others’ mental states. Indeed, the Intuitive Understanding factor correlated only weakly with the IRI perspective taking scale that has a stronger focus on effortful understanding.

However, it should be noticed that items in several relevant questionnaires (e.g., BEES, IRI, EQ [28,29,33,35]) actually lack items that explicitly measure Vicarious Experiences; they do not describe the experience of emotional states that are similar to those of another individual, but more general affective reactions. This may account for the fact that the obtained correlations were not particularly high. Upon detailed inspection, we suggest that the BEES and the IRI (empathic concern and personal distress) items might be closer to the sympathy construct. Only one recent questionnaire, the EAI [98,99] explicitly includes affective vicarious experiences, but it lacks the understanding component of empathy.

With respect to divergent validity, EES scores were weakly associated with depression and anxiety. This result is consistent with the study of Kim et al. [100] who investigated patients affected by a depressive disorder. The study evidenced differences between patients and healthy controls for all the dimensions of emotional intelligence, except for empathy [100]. The study also reported stability in empathy scores between the depressive episode and the remission phase. Furthermore, personal distress was moderately associated with cognitive anxiety (*r* = 0.45). The association between personal distress and depression or anxiety is in line with studies that reported an increase in personal distress in different groups of psychiatric disorders (e.g., schizophrenia, obsessive-compulsive disorder, Asperger syndrome, and fronto-temporal dementia [101–104]), or in people with difficulties in regulating their emotional states [105,106]. People who experience their emotions as excessively intense could be prone to experience personal distress, because they feel too much stimulated when they empathize with others’ negative emotions [107].

### Reliability

In our samples, internal consistency was satisfactory for the EES (McDonald’s omega > 0.90) [79], but not sufficient for the administered convergent measures of empathy (BEES and IRI; see S1 Text). The latter finding is consistent with previous studies [50,108] and is probably associated with the difficulties encountered by previous studies in replicating the original factor structures of these instruments [49–57].

Correlations with the MC-SDS (*p* < 0.20) indicated that responses to the EES are not significantly affected by social desirability. By contrast, convergent measures had in most cases significant (*p* < 0.05) and even moderate correlations with social desirability (*r* of 0.32 for BEES scores, and 0.35 for IRI empathic concern). This represents further support in favor of the EES in terms of reliability.

### Strengths and limitations

Some limitations of our study have to be mentioned. For example, we administered only two convergent measures of empathy (IRI and BEES). However, these were chosen, since they are the most used empathy measurements in international research. Secondly, our studies did not include adolescents and included only a small number of older adults. Thirdly, analyses indicated the presence of DIF as an effect of sex for few Vicarious Experience (N = 4) as well as for some Intuitive Understanding items (N = 3) suggesting that these items have different weights for males and females. Future research should investigate whether differential item functioning is detected in other independent samples and groups. Notwithstanding, the analyses also showed invariance of the factor structure of the EES across males and females, thus confirming that the two-factor model is adequate for both groups.

In spite of these limitations, our research has several methodological strengths. Firstly, we devised a new pool of items assessing emotional empathy and did not include items from previous questionnaires. Secondly, we reported results from two studies with very large and independent samples, and the wide age range of the included participants adds additional strength. Thirdly, we used up-to-date statistical analysis to assess the factor structure of the EES. Fourthly, the Vicarious Experience and Intuitive Understanding dimensions are measured for a broad range of emotions, including dysphoric and positive emotions, as well as generic emotional states (e.g., “Often, I am able to understand how people feel even before they tell me”). Fiftly, the respondent is asked to rate either his/her personal judgement or significant others’ judgement about his/her empathic response.

### Conclusions

Overall, our results indicate that the EES could be considered a reliable and valid assessment of empathy. Although mostly showing the expected convergence with relevant existing scales of empathy, the moderate correlations suggest that this scale could add valuable new information to the assessment of the empathy construct that is not captured by the other scales. Different from existing empathy scales, we approached empathy as intersubjective understanding, which provides a common theoretical basis for both experiential and cognitive aspects of empathy. In particular, the EES separately measures two dimensions of empathy that are relatively ignored or not combined in existing empathy questionnaires: the predisposition of the observer to participate in the emotions of someone else (Vicarious Experience), and his/her predisposition to consciously and intuitively grasp the emotional state of someone else (Intuitive Understanding). The propensities to vicariously experience and to understand others’ emotions can be considered fundamental to respond properly to others’ emotional states. Such responses can be expressed both at the level of affective responses, also known as sympathy [5,70,109] or empathic concern [29], and at the level of prosocial behavior by providing support to others [110].

The present findings further suggest that Vicarious Experience and a basic Intuitive Understanding of others’ emotional states could reflect distinguishable components of empathy, and that empathy might not be measured as a single construct. Further work is recommended to confirm the structural invariance of the scale, and to clarify the interrelatedness of Vicarious Experience and Intuitive Understanding, for instance, in terms of complementary, excitatory and inhibitory processes during social interaction.

## Supporting information

S1 Text Supporting information EES Text: Descriptions of the additional questionnaires. (DOCX)

S1 Table Supporting information EES Table 1: Factor loading of items in the construction sample. (DOCX)

S2 Table Supporting information EES Table 2: Standardized Factor loadings (λ). (DOCX)

S3 Table Supporting information EES Table 3: Correlations among the convergent empathy measures (Study 2). (DOCX)

S1 Questionnaire Supporting information EES Questionnaire Italian. (DOCX)

S2 Questionnaire Supporting information EES Questionnaire English. (DOCX)
