# Supplementary material for: A bidimensional measure of empathy: Empathic Experience Scale
Source: PLoS One. 2019 Apr 29;14(4):e0216164. doi: 10.1371/journal.pone.0216164 (PMC6488069; doi:10.1371/journal.pone.0216164)
Supplement: S1 Text — (DOCX) [file pone.0216164.s001.docx]

**SUPPORTING INFORMATION (S1 TEXT)**

**A Bidimensional Measure of Emotional Empathy: Empathic Experience Scale**

Marco Innamorati, Sjoerd J.H. Ebisch, Vittorio Gallese, Aristide Saggino

*Including: Supplementary text, 2 supplementary tables, the Empathic Experience Scale (EES) in Italian, English translation of the Empathic Experience Scale (EES)*

**Description of the additional questionnaires**

The IRI is a 28-items scale with a 5-point Likert type format for answering (from 1 = doesn’t describe me at all to 5 = describes me very well). Davis reported a four-factors structure ([Davis, 1980](#_ENREF_9), [1983](#_ENREF_10)): (1) Perspective taking (PT), assesses attempts to adopt the perspectives of other people; (2) Fantasy (FS), assesses the tendency to identify with fictional situations; (3) Empathic Concern (EC), assesses whether the individual has the tendency to experience compassionate feelings toward people in distress; and (4) Personal Distress (PD), assesses the personal feelings of discomfort when the individual is observing another’s negative experiences or is facing distressing situations. Cronbach alpha in this study were 0.78, 0.72, 0.75, and 0.80, respectively for FS, EC, PT, and PD.

The BEES is a 30-items questionnaire measuring emotional empathy. In the Italian version, each item is rated on a 7-point Likert type scale ranging from -3 to +3 ([Meneghini, Sartori, & Cunico, 2012](#_ENREF_14)). [Meneghini, Sartori, and Cunico (2006](#_ENREF_13)) investigated the factor structure of the Italian version of the BEES and reported five dimensions: (1) Impermeability to others people's emotional states (F1), consisting of 7 items assessing a difficulty in empathizing; (2) Susceptibility to other people's emotional states (F2), consisting of 6 items assessing the distress and participation for the emotional state of people who suffer or show positive emotions; (3) Diffuse emotional responsiveness (F3), consisting of 7 items assessing the tendency to respond emotionally to fictional situations (identifying oneself with fictional characters); (4) Susceptibility to contagion from people in one’s relational environment (F4), consisting of 6 items assessing emotional contagion in situations where another person is distressed or is suffering; and (5) Tendency to not letting oneself being involved in the distress or emotions of frail subjects (F5), consisting of 4 items assessing the difficulty of getting involved in elderly and children's feelings. Cronbach alpha values in this study were 0.69, 0.77, 0.74, 0.77, and 0.65, for F1, F2, F3, F4, and F5, respectively.

The original version of the MC-SDS is a 33 dichotomous items questionnaire measuring social desirability independently from psychopathology ([MC-SDS; Crowne & Marlowe, 1960](#_ENREF_8)). From the original version of the questionnaire, several brief versions of the MC-SDS have been produced in the last decades, differing in the number of items included ([Ballard, 1992](#_ENREF_3); [Reynolds, 1982](#_ENREF_15)). In our study, we used the 9-items Italian version of the MC-SDS ([Saggino & Perfetti, 2003](#_ENREF_16)). Cronbach alpha in the present sample was 0.68.

The TDI is a 21-items self-report instrument designed to assess major depressive disorder as specified by the latest editions of the Diagnostic and Statistical Manual of Mental Disorders (DSM; [American Psychiatric Association, 2000](#_ENREF_1), [2013](#_ENREF_2)), in order to overcome psychometric weaknesses of existing measures of depression ([Balsamo & Saggino, 2007](#_ENREF_7)). Each item is rated on a five-point Likert-type scale, ranging from 0 (always) to 4 (never). The TDI demonstrated strong psychometric properties ([Balsamo, Giampaglia, & Saggino, 2014](#_ENREF_5); [Balsamo, Innamorati, Van Dam, Carlucci, & Saggino, 2015](#_ENREF_6); [Ergun et al., 2016](#_ENREF_11); [Innamorati et al., 2013](#_ENREF_12)). In the present sample, Cronbach’s α was 0.88.

The STICSA-trait is a 21-items measure designed to assess cognitive (e.g. “I feel agonized over my problems,” “I think that others won’t approve of me”) and somatic (e.g. “My heart beats fast,” “My muscles are tense”) dimensions of trait anxiety. The individual rates how often a statement is true in general on a four-point Likert-type scale from 1 (not at all) to 4 (very much so). Psychometric properties of the Italian version of the STICSA have been investigated in a sample of older adults ([Balsamo, et al., 2015](#_ENREF_6)) and in the general population ([Balsamo et al., 2016](#_ENREF_4)). In the present sample, Cronbach’s α were 0.79 and 0.82, respectively for the STICSA somatic and the STICSA cognitive dimensions.

**References**

American Psychiatric Association. (2000). *Diagnostic and statistical manual of mental disorders* (4th ed., text revision ed.). Washington, DC: American Psychiatric Association.

American Psychiatric Association. (2013). *Diagnostic and Statistical Manual of Mental Disorders* (5th ed.). Arlington, VA: American Psychiatric Association.

Ballard, R. (1992). Short forms of the Marlowe-Crowne Social Desirability Scale. *Psychological Reports, 71*(3 Pt 2), 1155-1160. doi: 10.2466/pr0.1992.71.3f.1155

Balsamo, M., Carlucci, L., Sergi, M. R., Romanelli, R., D’Ambrosio, I., Fairfield, B., . . . Saggino, A. (2016). A new measure for trait and state anxiety: the State Trait Inventory of Cognitive and Somatic Anxiety (STICSA). Standardization in an Italian population. *Psicoterapia Cognitiva e Comportamentale, 22*, 229-232.

Balsamo, M., Giampaglia, G., & Saggino, A. (2014). Building a new Rasch-based self-report inventory of depression. *Neuropsychiatric Disease and Treatment, 13*, 153-165.

Balsamo, M., Innamorati, M., Van Dam, N. T., Carlucci, L., & Saggino, A. (2015). Measuring anxiety in the elderly: psychometric properties of the state trait inventory of cognitive and somatic anxiety (STICSA) in an elderly Italian sample. *International Psychogeriatrics, 27*(6), 999-1008. doi: 10.1017/S1041610214002634

Balsamo, M., & Saggino, A. (2007). Test per l’assessment della depressione nel contesto italiano: un’analisi critica. [Tests for the assessment of depression in Italian Context: a critical review]. *Psicoterapia Cognitiva e Comportamentale, 13*, 167-199.

Crowne, D. P., & Marlowe, D. (1960). A new scale of social desirability independent of psychopatology. *Journal of Consulting Psychology, 24*, 349-354.

Davis, M. H. (1980). A multidimensional approach to individual differences in empathy. *JSAS Catalog of Selected Documents in Psychology, 10*, 85.

Davis, M. H. (1983). Measuring individual differences in empathy: evidence for a multidimensional approach. *Journal of Personality and Social Psychology, 44*, 113-126. doi: doi: 10.1037/0022-3514.44.1.113

Ergun, T., Seckin Gencosmanoglu, D., Karakoc-Aydiner, E., Salman, A., Tekin, B., Bulbul-Baskan, E., . . . Onsun, N. (2016). Prevalence of obesity in paediatric psoriasis and its impact on disease severity and progression. *The Australasian journal of dermatology*. doi: 10.1111/ajd.12491

Innamorati, M., Tamburello, S., Contardi, A., Imperatori, C., Tamburello, A., Saggino, A., & Balsamo, M. (2013). Psychometric properties of the Attitudes toward Self-Revised in Italian young adults. *Depression Research and Treatment*. doi: 10.1155/2013/209216

Meneghini, A. M., Sartori, R., & Cunico, L. (2006). Adattamento e validazione su campione italiano della Balanced Emotional Empathy Scale di A. Mehrabian. *Ricerche di Psicologia, 29*, 123-152.

Meneghini, A. M., Sartori, R., & Cunico, L. (2012). *Balanced Emotional Empathy Scale. Adattamento italiano*. Firenze: Organizzazioni Speciali.

Reynolds, W. M. (1982). Development of reliable and valid short forms of the Marlowe-Crowne Social Desirability Scale. *Journal of Clinical Psychology, 38*, 119–125. doi: 10.1002/1097-4679(198201)38:1<119::AID-JCLP2270380118>3.0.CO;2-I

Saggino, A., & Perfetti, B. (2003, July, 13-17). *The Marlowe-Crowne Social Desirability Scale: a hierarchical factor analysis on an Italian sample.* Paper presented at the Biennial Meeting of the International Society of the Study of Individual Differences, Graz, Austria.
