# Supplementary material for: A bidimensional measure of empathy: Empathic Experience Scale
Source: PLoS One. 2019 Apr 29;14(4):e0216164. doi: 10.1371/journal.pone.0216164 (PMC6488069; doi:10.1371/journal.pone.0216164)
Supplement: S1 Table — (DOCX) [file pone.0216164.s002.docx]

**S1 Table.** Factor loading of items in the construction sample (*n* = 920).

| **Item** | **F1**  **Intuitive**  **Understanding** | **F2**  **Vicarious Experience** |  | **Item** | **F1**  **Intuitive**  **Understanding** | **F2**  **Vicarious**  **Experience** |
| --- | --- | --- | --- | --- | --- | --- |
| **ITEM #1** | 0.07 | **0.47** |  | ITEM #39 | 0.14 | -0.18 |
| **ITEM #2** | **0.59** | -0.01 |  | ITEM #40 | 0.27 | 0.29 |
| ITEM #3 | 0.34 | -0.27 |  | ITEM #41 | 0.38 | 0.21 |
| **ITEM #4** | -0.08 | **0.63** |  | **ITEM #42** | -0.05 | **0.69** |
| ITEM #5 | 0.38 | 0.01 |  | **ITEM #43** | -0.02 | **0.68** |
| **ITEM #6** | 0.02 | **0.54** |  | **ITEM #44** | **0.72** | 0.01 |
| **ITEM #7** | 0.10 | **0.41** |  | ITEM #45 | 0.16 | -0.31 |
| **ITEM #8** | **0.68** | -0.04 |  | **ITEM #46** | **0.78** | -0.07 |
| **ITEM #9** | **0.42** | 0.13 |  | **ITEM #47** | **0.62** | 0.07 |
| ITEM #10 | 0.14 | 0.29 |  | ITEM #48 | 0.29 | -0.21 |
| **ITEM #11** | **0.63** | -0.10 |  | **ITEM #49** | 0.16 | **0.50** |
| **ITEM #12** | **0.59** | -0.05 |  | **ITEM #50** | **0.70** | 0.03 |
| ITEM #13 | 0.06 | 0.34 |  | ITEM #51 | 0.13 | -0.28 |
| **ITEM #14** | **0.62** | -0.06 |  | ITEM #52 | -0.13 | 0.33 |
| ITEM #15 | 0.22 | 0.37 |  | **ITEM #53** | **0.65** | 0.004 |
| ITEM #16 | 0.23 | 0.36 |  | ITEM #54 | -0.02 | 0.24 |
| **ITEM #17** | **0.61** | 0.04 |  | **ITEM #55** | -0.09 | **0.43** |
| ITEM #18 | 0.25 | 0.17 |  | **ITEM #56** | **0.79** | -0.10 |
| **ITEM #19** | 0.08 | **0.49** |  | **ITEM #57** | **0.42** | -0.05 |
| **ITEM #20** | **0.70** | 0.02 |  | **ITEM #58** | -0.07 | **0.54** |
| **ITEM #21** | **0.41** | -0.30 |  | **ITEM #59** | **0.50** | 0.09 |
| **ITEM #22** | 0.02 | **0.58** |  | **ITEM #60** | 0.01 | **0.47** |
| **ITEM #23** | **0.62** | 0.001 |  | **ITEM #61** | -0.07 | **0.56** |
| **ITEM #24** | **0.46** | -0.13 |  | **ITEM #62** | **0.80** | -0.02 |
| **ITEM #25** | -0.02 | **0.58** |  | **ITEM #63** | -0.06 | **0.58** |
| **ITEM #26** | **0.59** | 0.07 |  | **ITEM #64** | -0.01 | **0.60** |
| **ITEM #27** | 0.06 | **0.40** |  | **ITEM #65** | **0.63** | 0.05 |
| **ITEM #28** | -0.04 | **0.56** |  | **ITEM #66** | **0.41** | -0.30 |
| **ITEM #29** | **0.67** | -0.02 |  | **ITEM #67** | 0.08 | **0.55** |
| **ITEM #30** | 0.05 | **0.60** |  | **ITEM #68** | **0.67** | 0.07 |
| ITEM #31 | 0.26 | 0.37 |  | **ITEM #69** | -0.03 | **0.61** |
| **ITEM #32** | **0.51** | 0.17 |  | **ITEM #70** | -0.01 | **0.64** |
| ITEM #33 | 0.31 | 0.04 |  | **ITEM #71** | **0.67** | 0.03 |
| **ITEM #34** | -0.03 | **0.71** |  | ITEM #72 | 0.14 | -0.34 |
| **ITEM #35** | **0.57** | 0.12 |  | **ITEM #73** | -0.10 | **0.68** |
| **ITEM #36** | -0.13 | **0.72** |  | **ITEM #74** | **0.43** | 0.15 |
| ITEM #37 | 0.14 | 0.34 |  | **ITEM #75** | -0.07 | **0.48** |
| **ITEM #38** | **0.49** | 0.21 |  |  |  |  |

Footnote. In bold items with loading ≥ 0.40.
