# Supplementary material for: A bidimensional measure of empathy: Empathic Experience Scale
Source: PLoS One. 2019 Apr 29;14(4):e0216164. doi: 10.1371/journal.pone.0216164 (PMC6488069; doi:10.1371/journal.pone.0216164)
Supplement: S2 Table — (DOCX) [file pone.0216164.s003.docx]

**S2 Table.** Standardized Factor loadings (λ).

| **Intuitive Understanding** | **Λ** | **Vicarious**  **Experience** | **Λ** |
| --- | --- | --- | --- |
| Item 2 | 0.73 | Item 1 | 0.72 |
| Item 4 | 0.71 | Item 3 | 0.59 |
| Item 6 | 0.72 | Item 5 | 0.67 |
| Item 8 | 0.76 | Item 7 | 0.54 |
| Item 10 | 0.75 | Item 9 | 0.70 |
| Item 12 | 0.82 | Item 11 | 0.78 |
| Item 14 | 0.79 | Item 13 | 0.76 |
| Item 16 | 0.68 | Item 15 | 0.76 |
| Item 18 | 0.81 | Item 17 | 0.77 |
| Item 20 | 0.72 | Item 19 | 0.65 |
| Item 22 | 0.77 | Item 21 | 0.58 |
| Item 24 | 0.83 | Item 23 | 0.59 |
| Item 26 | 0.78 | Item 25 | 0.70 |
| Item 28 | 0.78 | Item 27 | 0.75 |
| Item 30 | 0.67 | Item 29 | 0.74 |
