# Supplementary material for: A bidimensional measure of empathy: Empathic Experience Scale
Source: PLoS One. 2019 Apr 29;14(4):e0216164. doi: 10.1371/journal.pone.0216164 (PMC6488069; doi:10.1371/journal.pone.0216164)
Supplement: S3 Table — (DOCX) [file pone.0216164.s004.docx]

**S3 Table.** Correlations among the convergent empathy measures (Study 2)

Footnotes: BEES: Balanced Emotional Empathy Scale; IRI: Interpersonal Reactivity Index; SP: BEES Positive items; SN: BEES Negative items; FS: Fantasy Scale; EC: Empathic Concern; PT: Perspective Taking; PD: Personal Distress. Empathy is associated with lower scores on the BEES F1, F3, and F5 and higher scores on the BEES F2 and F4.
