# Supplementary material for: A bidimensional measure of empathy: Empathic Experience Scale
Source: PLoS One. 2019 Apr 29;14(4):e0216164. doi: 10.1371/journal.pone.0216164 (PMC6488069; doi:10.1371/journal.pone.0216164)
Supplement: S1 Questionnaire — (DOCX) [file pone.0216164.s005.docx]

**S1 Questionnaire (Italian version EES)**

**Istruzioni.** Legga attentamente le affermazioni che seguono e le valuti in base a quanto descrivono correttamente come lei normalmente si sente. Usi la scala che segue. Non si soffermi molto sulle singole domande e cerchi di rispondere il più sinceramente possibile.

| Per nulla vero | Poco vero | Abbastanza vero | Molto vero | Assolutamente vero  vero  vero |
| --- | --- | --- | --- | --- |
|  |  |  |  |  |
| 1 | 2 | 3 | 4 | 5 |

|  |  | **Per nulla vero** | **Poco vero** | **Abbastanza vero** | **Molto vero** | **Assolutamente vero** |
| --- | --- | --- | --- | --- | --- | --- |
|  | Mentre vedo un amico piangere sento i miei occhi inumidirsi. | □1 | □2 | □3 | □4 | □5 |
|  | Riesco spesso a capire come si sentono le persone anche prima che me lo dicano. | □1 | □2 | □3 | □4 | □5 |
|  | Le scene dei film dove il personaggio principale piange, perché tutto si è sistemato per il meglio, mi fanno venire le lacrime per la gioia. | □1 | □2 | □3 | □4 | □5 |
|  | Di solito mi accorgo subito quando un amico è arrabbiato. | □1 | □2 | □3 | □4 | □5 |
|  | Quando vedo in un film qualcuno che viene ferito, è come se sentissi quel dolore anche io. | □1 | □2 | □3 | □4 | □5 |
|  | Di solito riesco a capire “a pelle” quando i miei amici sono spaventati. | □1 | □2 | □3 | □4 | □5 |
|  | Quando vedo qualcuno che si pesta un dito con il martello, provo dolore anche io. | □1 | □2 | □3 | □4 | □5 |
|  | Quando qualcuno si sente preoccupato, riesco a capire intuitivamente il suo stato d’animo. | □1 | □2 | □3 | □4 | □5 |
|  | Vedendo un film drammatico provo la stessa tristezza dei personaggi dei film. | □1 | □2 | □3 | □4 | □5 |
|  | Capisco intuitivamente se qualcuno cui voglio bene è arrabbiato. | □1 | □2 | □3 | □4 | □5 |
|  | Vedere un adulto piangere per il dolore mi fa subito inumidire gli occhi. | □1 | □2 | □3 | □4 | □5 |
|  | Capisco subito se una persona è arrabbiata per qualcosa che gli è successo. | □1 | □2 | □3 | □4 | □5 |
|  | Quando vedo qualcuno che si fa male, il suo dolore lo vivo come mio senza possibilità di distanziarmene. | □1 | □2 | □3 | □4 | □5 |
|  | So capire intuitivamente come si sentono le persone cui voglio bene. | □1 | □2 | □3 | □4 | □5 |
|  | Chi mi conosce mi dice che non riesco a distanziarmi dalla tristezza degli altri. | □1 | □2 | □3 | □4 | □5 |
|  | Durante una telefonata riesco ad avvertire la tensione nella voce di una persona che conosco appena inizia a parlare. | □1 | □2 | □3 | □4 | □5 |
|  | Chi mi conosce mi dice che sono contagiato dalle emozioni degli altri. | □1 | □2 | □3 | □4 | □5 |
|  | Posso intuire velocemente lo stato d’animo di una persona che conosco anche se cerca di nascondere le sue reali emozioni. | □1 | □2 | □3 | □4 | □5 |
|  | I miei genitori pensano che vedere un parente che piange mi sconvolga completamente. | □1 | □2 | □3 | □4 | □5 |
|  | Riesco intuitivamente a capire che una persona si sente a disagio anche quando mi trovo in un gruppo di persone. | □1 | □2 | □3 | □4 | □5 |
|  | Quando qualcuno vicino a me ha un soprassalto per un rumore improvviso, sento immediatamente che il mio cuore comincia a battere all’impazzata. | □1 | □2 | □3 | □4 | □5 |
|  | Il mio intuito mi aiuta a capire se qualcuno è arrabbiato. | □1 | □2 | □3 | □4 | □5 |
|  | Mi sento eccitato quando vedo l’eccitazione di qualcun altro per qualcosa che gli è capitato. | □1 | □2 | □3 | □4 | □5 |
|  | Sono bravo a capire intuitivamente i sentimenti degli altri. | □1 | □2 | □3 | □4 | □5 |
|  | Quelli che vengono con me al cinema mi dicono che sono completamente preso dalle emozioni dei personaggi del film. | □1 | □2 | □3 | □4 | □5 |
|  | Mi accorgo subito se qualcuno in un gruppo è a disagio. | □1 | □2 | □3 | □4 | □5 |
|  | Sentire parlare di qualcuno addolorato per una perdita mi fa immediatamente sentire triste. | □1 | □2 | □3 | □4 | □5 |
|  | Mi dicono che sono bravo a capire intuitivamente le emozioni delle persone anche se qualcuno sta provando a celarle. | □1 | □2 | □3 | □4 | □5 |
|  | Se qualcuno prova angoscia, il mio cuore immediatamente comincia a battere veloce. | □1 | □2 | □3 | □4 | □5 |
|  | Comprendo velocemente se una persona cui voglio bene è felice. | □1 | □2 | □3 | □4 | □5 |

Scoring: il punteggio per tutti gli item è da sommare direttamente, non ci sono item da invertire.

Le due dimensioni sono:

Comprensione Intuitiva: item pari

Esperienza Vicaria: item dispari
