# Supplementary material for: A bidimensional measure of empathy: Empathic Experience Scale
Source: PLoS One. 2019 Apr 29;14(4):e0216164. doi: 10.1371/journal.pone.0216164 (PMC6488069; doi:10.1371/journal.pone.0216164)
Supplement: S2 Questionnaire — (DOCX) [file pone.0216164.s006.docx]

**S2 Questionnaire (English translation EES)**

**Instructions.** Please read very carefully the following statements and rate how strongly they describe how you normally feel. Use the following scale. Do not linger too much on the single statements and answer as sincerely as you can.

| Not at all true | Slightly true | Somewhat | Very true | Completely |
| --- | --- | --- | --- | --- |
|  |  | True |  | true |
| 1 | 2 | 3 | 4 | 5 |

|  |  | **Not at all true** | **Slightly true** | **Somewhat true** | **Very true** | **Completely true** |
| --- | --- | --- | --- | --- | --- | --- |
|  | While I see a friend crying, I feel myself getting teary-eyed. | □1 | □2 | □3 | □4 | □5 |
|  | Often, I am able to understand how people feel even before they tell me. | □1 | □2 | □3 | □4 | □5 |
|  | Scenes in movies where the main character cries because everything turns out for the best, give me tears of joy. | □1 | □2 | □3 | □4 | □5 |
|  | When a friend is angry, I am usually aware of it right away. | □1 | □2 | □3 | □4 | □5 |
|  | When I see someone get hurt in a movie, it’s as if I can feel the pain too. | □1 | □2 | □3 | □4 | □5 |
|  | I am usually able to understand instinctively when my friends are scared. | □1 | □2 | □3 | □4 | □5 |
|  | When I see someone hit his/her finger with a hammer, I feel pain too. | □1 | □2 | □3 | □4 | □5 |
|  | When someone feels worried, I am able to understand intuitively that person’s state of mind. | □1 | □2 | □3 | □4 | □5 |
|  | When I watch dramatic movies, I feel the same sadness as the characters in the story. | □1 | □2 | □3 | □4 | □5 |
|  | I know intuitively if someone that I love is angry. | □1 | □2 | □3 | □4 | □5 |
|  | Seeing an adult cry because of pain makes me suddenly get teary-eyed. | □1 | □2 | □3 | □4 | □5 |
|  | I know right away if someone is angry about something that happened to him/her. | □1 | □2 | □3 | □4 | □5 |
|  | When I see someone get hurt, I feel his/her pain as if I were hurt, without being able to distance myself from the pain. | □1 | □2 | □3 | □4 | □5 |
|  | I can understand intuitively how the people I love feel. | □1 | □2 | □3 | □4 | □5 |
|  | Those who know me tell me that I am not able to distance myself from the sadness of others. | □1 | □2 | □3 | □4 | □5 |
|  | During a phone call, I am able to perceive the tension in the voice of a person I know as soon as he/she starts talking. | □1 | □2 | □3 | □4 | □5 |
|  | Those who know me tell me that I am very affected by the emotions of others. | □1 | □2 | □3 | □4 | □5 |
|  | I can quickly intuit the state of mind of a person I know even if he/she tries to hide his/her real emotions. | □1 | □2 | □3 | □4 | □5 |
|  | My parents think that seeing one of my relatives cry completely upsets me. | □1 | □2 | □3 | □4 | □5 |
|  | I am able to know intuitively that a person feels uncomfortable even when I am in a group of people. | □1 | □2 | □3 | □4 | □5 |
|  | When someone standing close to me jumps suddenly because of an unexpected noise, I immediately feel my heart start beating excitedly. | □1 | □2 | □3 | □4 | □5 |
|  | My intuition helps me to understand if someone is angry. | □1 | □2 | □3 | □4 | □5 |
|  | When I see another person excited because of something that happened to him/her, I feel excited myself. | □1 | □2 | □3 | □4 | □5 |
|  | I am good at intuitively understanding the feelings of others. | □1 | □2 | □3 | □4 | □5 |
|  | People that come with me to the movie theater tell me that I get completely involved with the emotions of the characters in the movie. | □1 | □2 | □3 | □4 | □5 |
|  | I notice immediately if someone in a group feels uncomfortable. | □1 | □2 | □3 | □4 | □5 |
|  | Hearing about someone grieving from a recent loss immediately makes me feel sad. | □1 | □2 | □3 | □4 | □5 |
|  | People tell me that I am good at intuitively understanding the emotions of others even if someone is trying to hide his/her emotions. | □1 | □2 | □3 | □4 | □5 |
|  | If someone feels anguish, my heart immediately starts beating quickly. | □1 | □2 | □3 | □4 | □5 |
|  | I can understand right away if a person I love is happy. | □1 | □2 | □3 | □4 | □5 |

**Scoring:** Please sum up odd items scores for Vicarious Experience and even items for Intuitive Understanding
